# *Moringa oleifera* Seeds Improve Aging-Related Endothelial Dysfunction in Wistar Rats

**DOI:** 10.1155/2019/2567198

**Published:** 2019-05-13

**Authors:** Joseph Iharinjaka Randriamboavonjy, Sandrine Heurtebise, Pierre Pacaud, Gervaise Loirand, Angela Tesse

**Affiliations:** ^1^L'institut du thorax, INSERM, CNRS, UNIV Nantes, Nantes, France; ^2^L'institut du thorax, CHU Nantes, Nantes, France

## Abstract

Vascular aging is characterized by functional and structural changes of the vessel wall, including endothelial dysfunction, with decreased endothelial NO^·^ bioavailability and elevated vasoconstrictor and inflammatory mediator production, vascular rigidity, and tone impairment. *Moringa oleifera* (MOI) is a little tree, and different parts of which are used in traditional medicine in tropical Africa, America, and Asia for therapeutic applications in several disorders including cardiovascular disease. The present study is aimed at assessing the effect of MOI on aging-associated alteration of the endothelial function in Wistar rats. Middle-aged Wistar rats (46-week-old males) have been fed with food containing or not 750 mg/kg/day of MOI seed powder for 4 weeks. A group of young Wistar rats (16-week-old) was used as control. Measurement of isometric contraction, western blot analysis, and immunostaining has then been performed in the aortas and mesenteric arteries to assess the endothelium function. MOI treatment improved carbachol-induced relaxation in both aortas and mesenteric arteries of middle-aged rats. In the aortas, this was associated with an increased Akt signalling and endothelial NO synthase activation and a downregulation of arginase-1. In the mesenteric arteries, the improvement of the endothelial-dependent relaxation was related to an EDHF-dependent mechanism. These results suggest a vascular protective effect of MOI seeds against the vascular dysfunction that develops during aging through different mechanisms in conductance and resistance arteries.

## 1. Introduction

Vascular aging corresponds to functional and structural changes of the arterial wall characterized by endothelial dysfunction and vascular rigidity [1, 2]. A primary mechanism responsible for aging-induced endothelial dysfunction is the decrease in the bioavailability and the production of nitric oxide (NO^·^), mainly resulting from the increased oxidative stress. NADPH-derived superoxide anions (O_2_^−^) interact with NO to form peroxynitrite (ONOO^−^), which induces damaged protein accumulation and vascular inflammation and remodelling [3]. Peroxynitrite oxidizes the endothelial NO synthase (eNOS) cofactor BH4, thereby inactivating eNOS and reducing NO^·^ production [4, 5]. Moreover, BH4 reduction can result in eNOS uncoupling, leading to the generation of ROS rather than NO^·^ and thus perpetuating endothelial dysfunction [5]. Increased arginase expression and activity are also involved in endothelial dysfunction in aged vessels [6, 7]. Since arginase and eNOS compete for their common L-arginine (L-Arg) substrate, a rise in arginase activity or expression limits endothelial NO^·^ production in the vasculature [6–8]. Furthermore, the increased methylation of L-Arg and the resulting production of dimethyl L-Arg, observed in aged rats and humans, also contribute to the inhibition of eNOS activity [9].

In addition to decreased NO^·^ production and bioavailability, aging-associated endothelial dysfunction in resistance arteries also comprised oxidative stress-dependent impairment of prostacyclin and EDHF production that contributes to the defective endothelium-mediated vasorelaxation [5, 10].

Several studies suggested that polyphenols and other natural compounds contained in bioactive extracts and foods can protect against vascular aging through their anti-inflammatory and antioxidant properties as well as for their ability to improve eNOS activity and NO^·^ bioavailability [11–13]. *Moringa oleifera* (MOI) is a little tree used in Malagasy traditional medicine to treat several pathological states such as hypertension and inflammation [14, 15]. MOI seed oil showed free radical scavenging activity due to molecules such as flavonoids known to have antioxidant properties [16]. We previously described the cardiovascular protective effect of the oral administration of MOI seeds against cardiac complications induced by high blood pressure (left ventricle hypertrophy and fibrosis), vascular inflammation, and oxidative stress in spontaneously hypertensive rats (SHR), thus showing the anti-inflammatory and antioxidant action of a diet containing MOI seeds [17, 18].

The aim of the present work was to investigate the potential beneficial effects of MOI seeds administrated during 4 weeks against established aging-related vascular dysfunction in middle-aged Wistar rats (MAWR) by analysing endothelial function in conductance (aorta) and resistance (mesenteric) arteries.

## 2. Methods

### 2.1. Animals

Male Wistar rats (46 weeks old, middle-aged) were divided into two groups: a group receiving normal food (MAWR) and a group fed with food containing MOI seed powder (750 mg/day) mixed with standard pellet diet (MOI MAWR) for 4 weeks. This dose is within the range of concentrations classically used in experimental rodent models [18, 19]. A control group of young rats (16 weeks old, YWR), receiving normal food, was also used to check that middle-aged rats did indeed have arterial dysfunction. All groups received water ad libitum. At the end of the experimental protocol, all the rats were euthanized. The thoracic aorta and mesenteric arteries were then collected for vascular reactivity, western blot, and immunohistological analyses. All experiments were conducted in agreement with our Ethical Committee *Guide for the Care and Use of Laboratory Animals* (authorisation number 00909.01).

### 2.2. Arterial Reactivity

The aortas and first branches of superior mesenteric arteries were collected in physiological saline solution (in mM; 130 NaCl, 5.6 KCl, 1 MgCl_2_, 2 CaCl_2_, 11 glucose, and 10 Tris, pH 7.4 with HCl), cleaned, and cut in 2 mm long rings. Arterial rings were then mounted on multichannel isometric myograph, bathed in Krebs-Henseleit solution at 37°C bubbled with 95% O_2_-5% CO_2_, and connected to a force transducer (Pioden Controls Ltd., Canterbury, UK, for aortic rings; Danish Myo Technology, Aarhus, Denmark, for mesenteric artery rings). After equilibration, the contractile response to KCl (60 mM) was measured. Endothelial function was tested by measuring the relaxing response to cumulative doses of carbachol (CCh, 1 nM-10 *μ*M, Sigma-Aldrich) of rings precontracted by phenylephrine (PhE, 1 *μ*M, Sigma-Aldrich) in the absence and presence of L-N^G^-nitroarginine methyl ester (L-NAME, 100 *μ*M, Sigma-Aldrich) alone or in association with the cyclooxygenase (COX) inhibitor, indomethacin (10 *μ*M, Sigma-Aldrich). Digital data were recorded by a MacLab/4e recorder and analysed using a LabChart v7 software (AD Instruments, Paris, France).

### 2.3. Staining and Confocal Microscopy Imaging

Frozen sections of the aortas (7 *μ*m thick) were fixed with cold 100% methanol and incubated for 2 h at room temperature in blocking buffer (5% of albumin in PBS). Tissue sections were then incubated overnight (4°C) with a mouse monoclonal antibody against the phosphorylated-(Ser 1179)-eNOS (1 : 50, Santa Cruz Biotechnology) or a rabbit polyclonal antibody against phosphorylated-(Ser473)-Akt (p-Akt) protein (1/500, Cell Signaling). Three washes were followed by incubation (1 h, at room temperature, in the dark) with the secondary mouse or rabbit fluorescent Alexa fluor-647-conjugated antibody (1 : 500, Molecular Probes). A Nikon A1-RS inverted laser scanning confocal microscope was used for the optical sectioning of the tissue. Digital image recording was performed using the NIS element software. Images were analysed and processed by Fiji software.

### 2.4. Western Blot Analysis

The aortas were homogenized and lysed. Proteins (50 *μ*g) were separated in precoated SDS-PAGE 4-15% (MiniPROTEAN® TGX™, Bio-Rad), transferred to nitrocellulose membrane, and then incubated (2 h at room temperature) in blocking buffer (5% nonfat dry milk in PBS). The membranes were probed overnight at 4°C with primary rabbit polyclonal antibody directed to arginase-1 (1/200, Santa Cruz Biotechnology). After 3 washes, immunoreactive bands were revealed with a secondary peroxidase-conjugated anti-rabbit IgG (1 : 5000, Beckman Coulter), detected by enhanced chemiluminescence system (ECL Plus, Amersham Biosciences), and quantified by densitometry. A polyclonal anti-*α*-tubulin antibody (1 : 5000, Sigma-Aldrich) was used to check protein gel loading and to normalize protein expression. The analysis of the blots was performed with Image Lab™ software v4.1 (Bio-Rad).

### 2.5. Data Analysis

The endothelial relaxation in response to cumulative doses of CCh was expressed as a percentage of the amplitude of PhE-induced precontraction. For the statistical analysis of these data, we used a two-way analysis of variance for repeated measures with subsequent Bonferroni post hoc test. To calculate the area under the curve (AUC) of the concentration-dependent relaxation in response to CCh, we used the GraphPad Prism 5.02 software. A one-way ANOVA with subsequent Bonferroni post hoc test or the ANOVA on ranks was performed for the AUC of relaxation and protein expression data. All statistical analysis were realized with SigmaStat 3.5 software. All values are presented as mean ± SD of *n* repetitive measurements, with *n* representing the number of rat samples. ^∗^ *p* < 0.05 was considered to be statistically significant.

## 3. Results

### 3.1. MOI Administration Restores CCh-Induced Relaxation in MAWR Aortas

**Figure 1:**
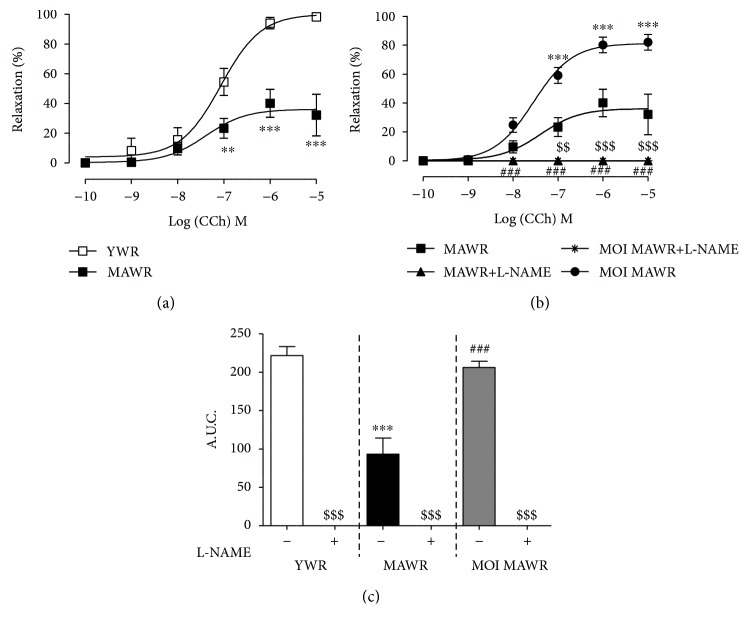
(a) Carbachol- (CCh-) induced relaxation in the aortas from young rats (YWR: 16 weeks old) and control middle-aged rats (MAWR, 50 weeks old; ^∗∗^ *p* < 0.01, ^∗∗∗^ *p* < 0.001, YWR versus MAWR). (b) Relaxation curves to CCh in the aortas from MAWR and MOI-treated middle-aged rats (MOI MAWR) in the absence and in the presence of L-NAME (100 *μ*M) (^∗∗∗^ *p* < 0.001, MOI MAWR versus MAWR; ^###^ *p* < 0.001, MAWR versus MAWR+L-NAME; ^$$^ *p* < 0.01, ^$$$^ *p* < 0.001, MOI MAWR versus MOI MAWR+L-NAME). (c) Histogram showing the area under the curve (AUC) of CCh-induced relaxation without and with L-NAME in YWR, MAWR, and MOI MAWR (^∗∗∗^ *p* < 0.001, MAWR versus YWR; ^###^ *p* < 0.001 MOI MAWR versus MAWR; ^$$$^ *p* < 0.001 without L-NAME versus with L-NAME in the same group. Results are expressed in mean ± SD; *n* = 5).

We first assessed the effect of MOI treatment on endothelial-dependent relaxation of aorta rings from MAWR by measuring the CCh-dependent relaxation in the three groups of rats and calculating the AUC in the absence and in the presence of L-NAME (Figure 1). As expected, CCh-induced endothelium-dependent relaxation was reduced in the aortas of MAWR compared to YWR aortas (Figures 1(a) and 1(c)). MOI seed administration almost completely restored CCh-induced relaxation in the aortas of MAWR that returned similar to that observed in YWR (Figures 1(b) and 1(c)). L-NAME completely abolished CCh-induced relaxation in the aortas from YWR, MAWR, and MOI MAWR (Figures 1(b) and 1(c) and Supplementary Figure 1) indicating that the relaxation response to CCh in the aorta is completely dependent of NO^·^ in the three groups of rats. These data suggest that the improvement of the endothelial function induced by MOI treatment in the aortas could result from the correction of aging-induced NO^·^ signalling defect.

### 3.2. MOI Administration Improves eNOS Signalling in the Aortas

**Figure 2:**
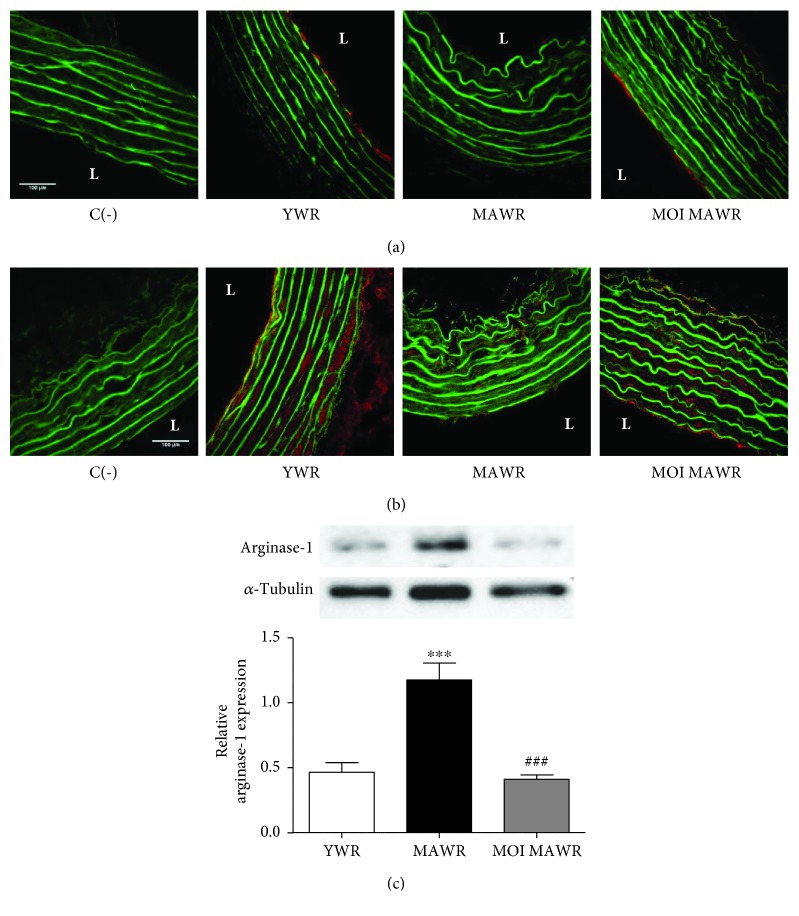
(a) Immunohistochemical representative red staining of phosphorylated-(Ser 1179)-eNOS or phosphorylated-(Ser 473)-Akt (b) in the aortas from YWR, control MAWR, and MOI MAWR. Green fluorescence corresponds to elastin autofluorescence. L = lumen of the vessel. C(-) is a negative control without the primary antibody. Scale bar = 100 *μ*m. (c) Representative western blot and corresponding densitometric analysis of arginase-1 expression normalized to *α*-tubulin in the aortas of YWR, MAWR, and MOI MAWR (mean ± SD, *n* = 3; ^∗∗∗^ *p* < 0.001 MAWR versus YWR; ^###^ *p* < 0.001 MOI MAWR versus MAWR).

To confirm the effect of MOI treatment on NO^·^ signalling, we directly assessed eNOS activity by immunostaining on cross sections of the aortas of active phosphorylated-(Ser1179)-eNOS and active phosphorylated-(Ser473)-Akt, one of the main kinases phosphorylating eNOS at Ser1179 [20] (Figures 2(a) and 2(b)). Both endothelial phosphorylated-(Ser1179)-eNOS and phosphorylated-(Ser473)-Akt staining observed in YWR were lost in MAWR but were restored in MOI MAWR (Figures 2(a) and 2(b)) suggesting a role of Akt-induced eNOS activation in the beneficial effect of MOI on endothelial function in MAWR. We next assessed the expression of arginase-1 that might also contribute to the reduced production of NO^·^ with aging [21] (Figure 2(c)). Arginase-1 expression, increased in MAWR aortas compared to YWR, was decreased to a level similar to YWR in MOI MAWR (Figure 2(c)). These data are consistent with a beneficial effect of MOI on eNOS activity in the aortas of MAWR.

### 3.3. MOI Administration Improves Endothelial Function in AWR Mesenteric Arteries

**Figure 3:**
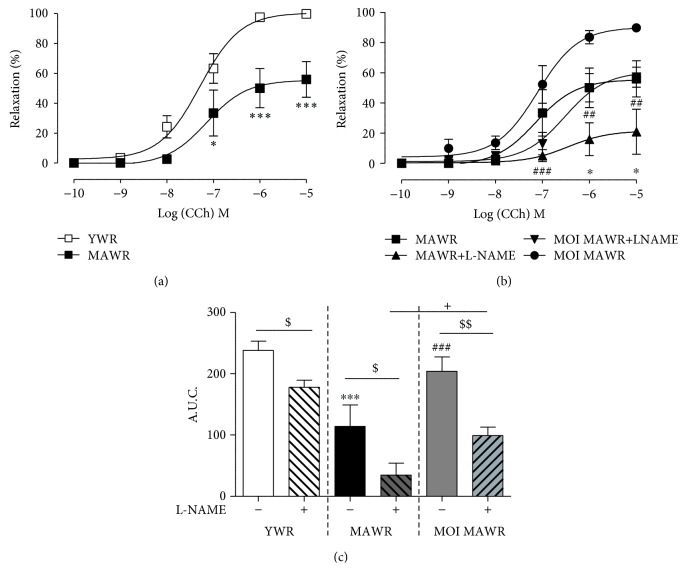
(a) Carbachol- (CCh-) induced relaxation in the mesenteric arteries of YWR and control MAWR (^∗^ *p* < 0.05, ^∗∗∗^ *p* < 0.001, MAWR versus YWR). (b) Relaxation curves to CCh in the mesenteric arteries from MAWR and MOI MAWR in the absence and in the presence of L-NAME (100 *μ*M) (^∗^ *p* < 0.05, MAWR+L-NAME versus MAWR; ^##^ *p* < 0.01, ^###^ *p* < 0.001, MOI MAWR+L-NAME versus MOI MAWR). (c) Histograms showing the area under the curve (AUC) without and with L-NAME for YWR, MAWR, and MOI MAWR (^∗∗∗^ *p* < 0.001, MAWR versus YWR; ^###^ *p* < 0.001, MOI MAWR versus MAWR; ^$^ *p* < 0.05, ^$$^ *p* < 0.01, without L-NAME versus with L-NAME in the same group; ^+^ *p* < 0.05 MOI MAWR+L-NAME versus MAWR+L-NAME). Results were expressed in mean ± SD of *n* = 5 rats.

We also assessed the effect of MOI on the endothelial function of resistance arteries from MAWR. Both the dose-dependent relaxation curve to CCh and the AUC show that the relaxation induced by CCh was decreased in MAWR mesenteric arteries compared to YWR vessels (Figures 3(a) and 3(c)). MOI seeds improved the endothelial relaxation in the mesenteric arteries of MAWR compared to nontreated MAWR (Figures 3(b) and 3(c)). In YWR, L-NAME induced only a rightward shift of the concentration-relaxation curve to CCh without change of the maximal relaxation, indicating that NO^·^ did not play a major role in CCh-induced relaxation of YWR mesenteric arteries (Supplementary Figure 2). Accordingly, the AUC was only decreased by ~25% by L-NAME in YWR mesenteric arteries (Figure 3(c)). In contrast, L-NAME strongly reduced the CCh-induced relaxation in MAWR as illustrated by the ~80% reduction of the maximal CCh-induced relaxation (Figure 3(b)) and the ~70% reduction of the AUC observed in the presence of L-NAME (Figure 3(c)). This suggests that aging increased the NO^·^ component of CCh-induced relaxation in the mesenteric arteries. MOI increased the relaxation response in MAWR mesenteric arteries, and L-NAME reduced only partially the CCh-induced relaxation in MOI MAWR, suggesting that the beneficial effect of MOI on the endothelial function was not mainly mediated by NO^·^ signalling but involved other mechanism(s) (Figures 3(b) and 3(c)).

### 3.4. MOI Administration Enhances EDHF-Dependent Relaxation in MAWR Mesenteric Arteries

**Figure 4:**
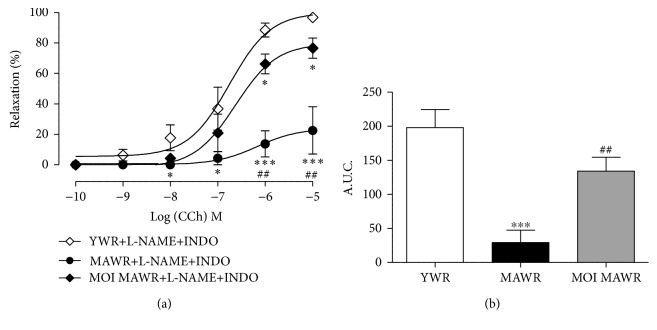
(a) EDHF-dependent relaxation to carbachol (CCh) in the mesenteric arteries from YWR, MAWR, and MOI MAWR obtained in the presence of L-NAME and indomethacin (INDO), L-NAME+INDO (^∗^ *p* < 0.05, ^∗∗∗^ *p* < 0.001, versus YWR+L-NAME+INDO). (b) Histograms showing the area under the curve (AUC) of CCh-dependent relaxation of YWR, MAWR, and MOI MAWR mesenteric artery in the presence of L-NAME and INDO (L-NAME+INDO) (^∗∗∗^ *p* < 0.001, MAWR+L-NAME+INDO versus YWR+L-NAME+INDO; ^##^ *p* < 0.01, MOI MAWR+L-NAME+INDO versus MAWR+L-NAME+INDO; results are expressed in mean ± SD with *n* = 5 per group).

We hypothesized that MOI improved endothelium-dependent relaxation in the mesenteric arteries of MAWR by increasing the contribution of EDHF. We therefore directly measured the EDHF-dependent component of the CCh-induced relaxation in the presence of both L-NAME and INDO to block both NO^·^- and COX-deriving relaxing factors, respectively (Figure 4). The EDHF-mediated relaxation of the mesenteric arteries in response to CCh was significantly decreased in MAWR compared to YWR, indicating that in mesenteric arteries CCh-induced relaxation was essentially dependent on EDHF in YWR and that aging strongly reduced this EDHF-mediated relaxation (Figures 4(a) and 4(b)). MOI not totally but strongly restored EDHF-mediated relaxation in MAWR (Figures 4(a) and 4(b)). These data thus indicate that the altered endothelium-dependent relaxation response to CCh in MAWR mesenteric arteries resulted from a loss of its EDHF-mediated component which was partially corrected by MOI.

## 4. Discussion

Our study provides evidence for the beneficial arterial effects of MOI seed administration in MAWR with an established aging-induced endothelial dysfunction in both the aorta and mesenteric arteries. Diet containing MOI seeds improves the endothelial function in MAWR by boosting eNOS activity in the aorta and enhancing EDHF-dependent relaxation in the mesenteric arteries. These data suggest a different effect of MOI seeds on the endothelial aging process in conductance and resistance arteries.

Vascular aging consists of a set of changes in the mechanical and structural properties of the vascular wall including a decrease in endothelium-dependent relaxation by reducing the bioavailability of NO^·^ and/or EDHF depending on the arterial bed [4, 10, 22]. As previously described [23, 24], we confirm that the endothelium-dependent CCh-induced relaxation in the aortas was exclusively mediated by NO^·^ both in YWR and MAWR. Aging was associated with a reduction of endothelium-dependent NO^·^ signalling attested by the loss of the CCh-induced relaxation and the decrease of phosphorylation of eNOS and its upstream kinase Akt, in agreement with the role of Akt/eNOS signalling impairment in aging-induced endothelial dysfunction [20, 25]. MOI administration corrected the Akt/eNOS defect and restored NO^·^-mediated endothelium-dependent relaxation in MAWR. NO signalling has been identified as a target of the beneficial action of polyphenol-rich diets on endothelial function and cardiovascular protection [26, 27]. Indeed, similar improvements of endothelial function by activating the Akt/eNOS pathway were previously demonstrated in a diet supplemented with other natural substances such as (R)-*α*-lipoic acid contained in several vegetal foods and other polyphenols such as delphinine and resveratrol contained in red wine [25, 28, 29]. Polyphenolic compounds contained in MOI seeds could thus be responsible for or participate in the beneficial effects of MOI seeds on Akt/eNOS signalling in the endothelium of MAWR [30, 31]. Indeed, MOI seeds contain glucosinolates (glucomoringin) and isothiocyanates known for their ability to improve endothelial-dependent relaxation in rat aortas [32].

In addition to the stimulation of Akt/eNOS signalling, we observed that MOI reduced arginase-1 expression which was upregulated in MAWR. This MOI-induced reduction of arginase-1 expression can therefore provide an additional mechanism by which MOI improves NO^·^ signalling, by increasing the amount of the eNOS substrate L-Arg required for NO^·^ biosynthesis. Targeting arginase is considered as an emergent strategy to elevate NO^·^ level in disorders involving endothelial dysfunction and thus as a potential target for the treatment of cardiovascular disease [33]. Several plant-derived substances, especially polyphenols, have been shown to inhibit arginase activity [34]. In particular, MOI leaf extracts inhibited arginase activity in a dose-dependent manner [35]. This effect has been ascribed to the polyphenols (gallic acid, catechin, chlorogenic acid, ellagic acid, epicatechin, rutin, quercitrin, isoquercitrin, quercetin, and kaempferol) identified in the extract. Here, we show that MOI seeds downregulate arginase-1 protein expression, suggesting that plant-derived polyphenols can reduce the deleterious effect of arginase-1 on endothelial NO^·^ signalling by inhibiting both its activity and its expression.

In the mesenteric arteries, MOI restored the EDHF-mediated CCh-induced relaxation that was decreased in MAWR. Although not demonstrated in this study, hydrogen peroxide (H_2_O_2_), known as the main hyperpolarizing factor in the mesenteric arteries, could be involved in the effect of MOI [10, 36]. Other mechanisms such as the upregulation of Ca^2+^-activated small (SK_Ca_), intermediate (IK_Ca_), and large (BK_Ca_) conductance potassium channels, Na^+^/K^+^ ATPase pump, and the myoendothelial gap junctions can be also involved in the NO-independent effect of MOI in the mesenteric arteries [37, 38]. Indeed, the improvement of aging-related endothelial dysfunction, particularly its EDHF component, produced by red wine polyphenolic compounds has been ascribed, at least in part, to the normalization of SK_Ca_ and IK_Ca_ expression, reduced in MAWR mesenteric arteries [10]. Similar to MOI, the beneficial effect of dietary supplementation with red wine polyphenolic compounds on endothelial dysfunction results from the potentialization of the EDHF-dependent relaxation in resistance arteries while it was due to the increase in NO^·^ bioavailability in conductance arteries [39]. Thus, the endothelial effect of dietary MOI on the EDHF pathway is probably also due to the polyphenolic compounds contained in MOI seeds, in agreement with the polyphenolic compound-related antioxidant and anti-inflammatory effects of a diet containing MOI seeds that we previously described [18]. Complementary experiments to measure plasma biomarkers of inflammation, oxidative stress, and endothelial dysfunction such as resistin, ICAM-1, VCAM-1, and E-selectin could be useful to refine the mechanism of action of MOI.

The beneficial effect of MOI on endothelium function has been observed in male MAWR. However, it has been described that male and female rodents are not equally prone to age-induced endothelial dysfunction [40]. It thus would be interesting to assess whether MOI seeds would also be able to improve endothelial function in female rats.

## 5. Conclusion

This study shows that dietary supplementation with MOI corrects aging-induced endothelial dysfunction. The beneficial effects of MOI seeds result from the upregulation of NO^·^ and EDHF signalling in the aorta and mesenteric artery, respectively. They are likely due to the polyphenolic compounds contained in MOI seeds. Our data suggest the interest of dietary supplementation with MOI seeds in middle-aged or elderly people to limit aging-related endothelial dysfunction and prevent the development of cardiovascular disease. This could be particularly relevant in MOI-producing countries that may have limited access to pharmacological treatments and also in Western countries, as a nondrug mean for healthy aging.

## Data Availability

The data of the vascular relaxation, the area under the curves of relaxation, and the values of protein expression (arginase-1) will be available on request asking directly the corresponding author writing a mail at angela.tesse@univ-nantes.fr.

## Abbreviations

MAWR: Middle-aged Wistar rats
CCh: Carbachol
COX: Cyclooxygenase
EDHF: Endothelium-derived hyperpolarizing factor
eNOS: Endothelial NO synthase
INDO: Indomethacin
iNOS: Inducible NO synthase
L-Arg: L-arginine
L-NAME: L-N^G^-nitroarginine methyl ester
MOI: *Moringa oleifera*
MOI MAWR: Middle-aged Wistar rats treated with *Moringa oleifera* seeds
NO: Nitric oxide
PHE: Phenylephrine
RAS: Renin/angiotensin system
ROS: Reactive oxygen species
RWPs: Red wine polyphenols
SOD: Superoxide dismutase
AUC: Area under the curve
YWR: Young Wistar rats.

## Supplementary Materials

Supplementary Materials Supplementary Figure 1: carbachol- (CCh-) induced relaxation in the aortas from young rats (YWR: 16 weeks old) in the absence and in the presence of L-NAME (100 *μ*M) (^∗∗∗^ *p* < 0.001, +L-NAME versus without L-NAME. Results are expressed in mean ± SD with *n* = 5 per group). Supplementary Figure 2: carbachol- (CCh-) induced relaxation in the mesenteric arteries from young rats (YWR: 16 weeks old) in the absence and in the presence of L-NAME (100 *μ*M) (^∗∗^ *p* < 0.01, +L-NAME versus without L-NAME; results are expressed in mean ± SD with *n* = 5 per group).
