# Supplementary material for: Moringa oleifera Seeds Improve Aging-Related Endothelial Dysfunction in Wistar Rats
Source: Oxid Med Cell Longev. 2019 May 13;2019:2567198. doi: 10.1155/2019/2567198 (PMC6535829; doi:10.1155/2019/2567198)
Supplement: Supplementary Materials — Supplementary Figure 1: carbachol- (CCh-) induced relaxation in the aortas from young rats (YWR: 16 weeks old) in the absence and in the presence of L-NAME (100 μM) (∗∗∗ p < 0.001, +L-NAME versus without L-NAME. Results are expressed in mean ± SD with n = 5 per group). Supplementary Figure 2: carbachol- (CCh-) induced relaxation in the mesenteric arteries from young rats (YWR: 16 weeks old) in the absence and in the presence of L-NAME (100 μM) (∗∗ p < 0.01, +L-NAME versus without L-NAME; results are expressed in mean ± SD with n = 5 per group). [file 2567198.f1.pptx]

## Slide 1
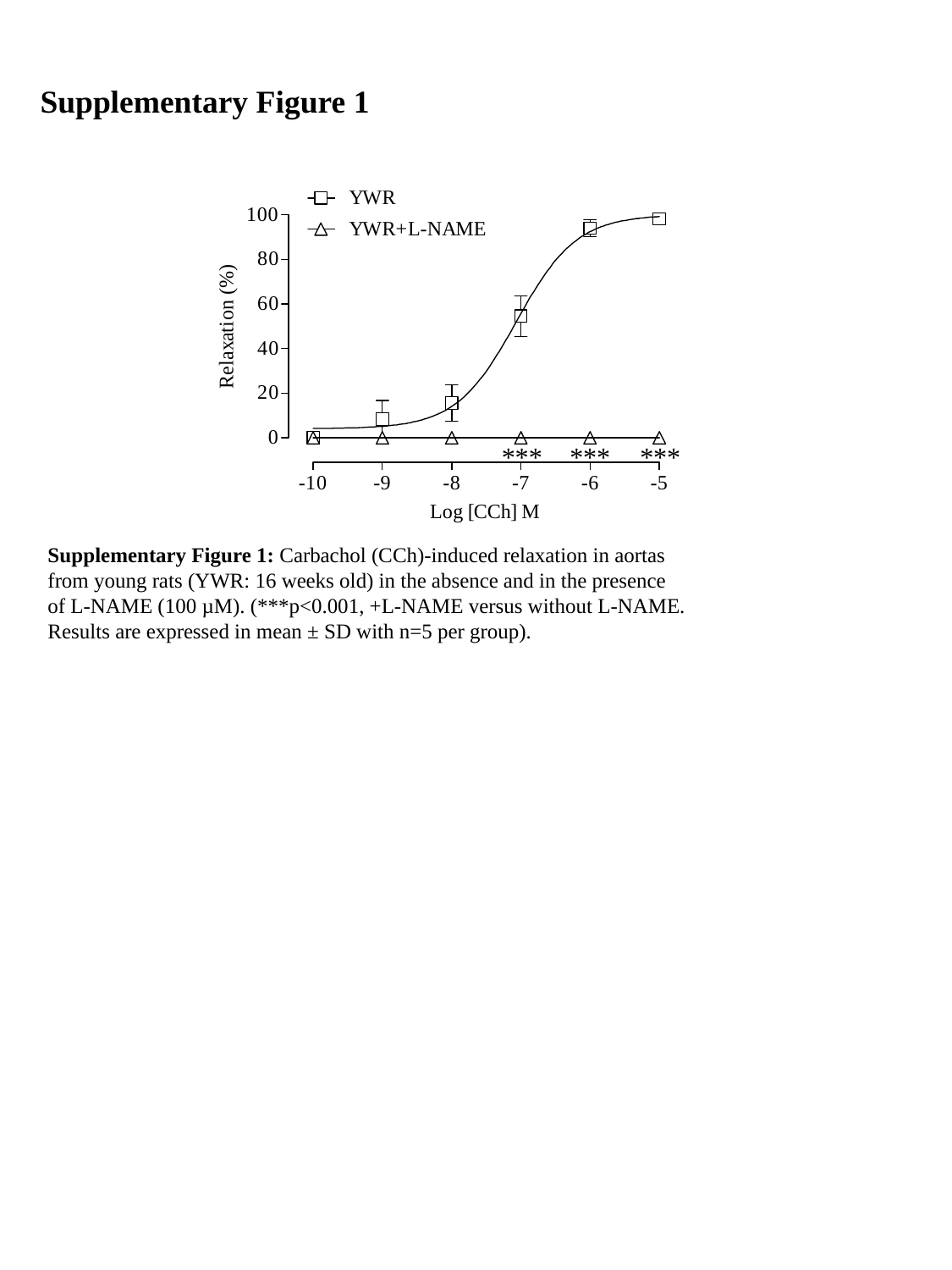

Supplementary Figure 1
Supplementary Figure 1: Carbachol (CCh)-induced relaxation in aortas
from young rats (YWR: 16 weeks old) in the absence and in the presence
of L-NAME (100 µM). (***p<0.001, +L-NAME versus without L-NAME.
Results are expressed in mean ± SD with n=5 per group).

## Slide 2
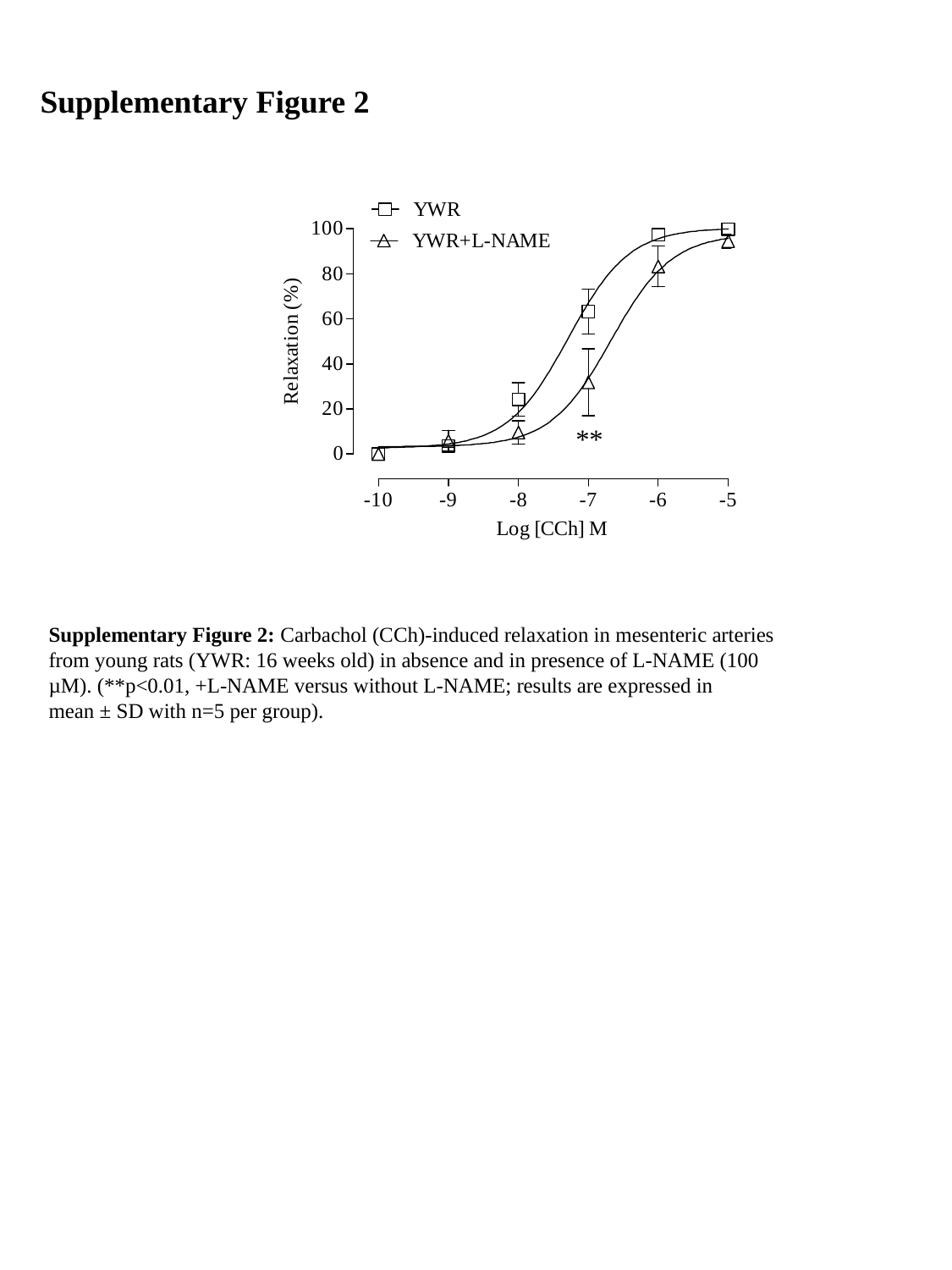

Supplementary Figure 2
Supplementary Figure 2: Carbachol (CCh)-induced relaxation in mesenteric arteries from young rats (YWR: 16 weeks old) in absence and in presence of L-NAME (100 µM). (**p<0.01, +L-NAME versus without L-NAME; results are expressed in
mean ± SD with n=5 per group).
